# Sustainable dyeing of ramie fiber with ternary reactive dye mixtures in liquid ammonia†

**DOI:** 10.1039/d2ra03288k

**Published:** 2022-07-01

**Authors:** Bo Gao, Xiaolong Huang, Tiancheng Jiang, Md. Nahid Pervez, Wenju Zhu, Mohammad Mahbubul Hassan, Yingjie Cai, Vincenzo Naddeo

**Affiliations:** Hubei Provincial Engineering Laboratory for Clean Production and High Value Utilization of Bio-based Textile Materials, Wuhan Textile University Wuhan 430200 China yingjiecai@wtu.edu.cn; College of Art and Design, Wuhan Textile University Wuhan 430200 China; Engineering Research Centre for Clean Production of Textile Dyeing and Printing, Ministry of Education, Wuhan Textile University Wuhan 430200 China; Sanitary Environmental Engineering Division (SEED), Department of Civil Engineering, University of Salerno via Giovanni Paolo II 132, 84084 Fisciano (SA) Italy vnaddeo@unisa.it; Fashion, Textiles and Technology Institute (FTTI), University of the Arts London 20 John Prince's Street London W1G 0BJ UK

## Abstract

Liquid ammonia (LA) dyeing is a zero-effluent and sustainable dyeing technology investigated for textiles. In the present work, three bi-functional reactive dyes, Reactive Red 195 (R195), Reactive Yellow 145 (Y145), and Reactive Blue 194 (B194), were used to dye ramie fiber in liquid ammonia, and the dye exhaustion (%) and fixation (%) were compared with ramie fibers dyed with the same dyes in an aqueous dyeing method. Dyeing with a single reactive dye, a binary dye mixture, and a ternary dye mixture in liquid ammonia showed that all the dyes are highly compatible as they showed similar uptake. The total dye exhaustion percentage of dyeing with the ternary dye mixture was 22.6%. After dyeing, a cationic fixing agent (CFA)/decamethylcyclopentasiloxane (D5) micro-emulsion was applied and the dye fixation rate was 96.7% accompanied by high colorfastness to washing (Grade 4–5) and produced uniform shades. Finally, a color triangle of dyed ramie fibers was prepared to exhibit many colorful shades. This work demonstrates the viability of dyeing of textile fibers in liquid ammonia.

## Abstract 2

Liquid ammonia (LA) dyeing is a zero-effluent and sustainable dyeing technology investigated for textiles.

## Graphical abstract

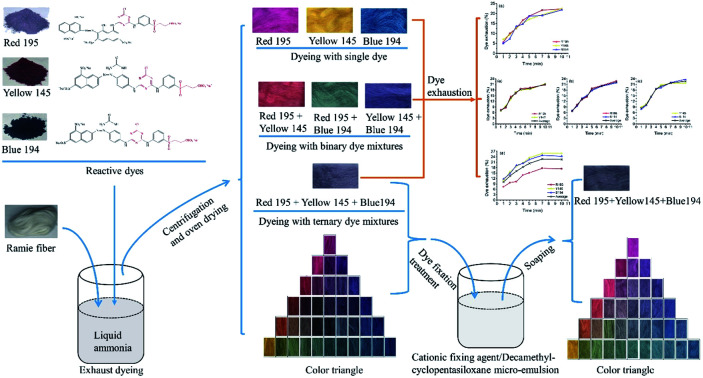

## 1. Introduction

Textile dyeing and finishing industries are considered one of the greatest industrial polluters because they discharge a large amount of wastewater containing harmful dyes affecting the environment and human life.^1,2^ In this regard, over the past several years, technologies such as adsorption,^3,4^ Fenton catalytic systems,^5,6^ and membranes^7–9^ have been applied to treat these complex wastewater effluents; however, these methods are still questionable in terms of sustainable development in the textile industry. Therefore, waterless dyeings, such as dyeing in supercritical fluid,^10–12^ silicone oil,^13–17^ liquid paraffin oil,^18–20^ plant oil,^21,22^ and ethanol,^23,24^ have been studied as an example of a cleaner dyeing method to reduce water pollution.

Liquid ammonia (LA) is a polar, transparent, and anhydrous medium, in which anionic dyes become soluble and thus it could be a potential medium for dyeing cellulosic fiber. In the LA dyeing of cellulosic fiber with reactive dye, the dye molecules are mixed with liquid ammonia at a molecular level without forming any dye aggregates. When the fiber is immersed in the LA dyebath, the fiber is wetted and swelled immediately by LA due to its low surface tension. Simultaneously, the air and moisture in the fiber are extruded, which benefits the migration of LA dyebath inside the fiber. The LA quickly damages the crystalline regions of the fiber and converts them to amorphous regions allowing quick penetration of dye molecules into the fiber.^25^ In the LA dyeing method, the dyes are only physically adsorbed into the fiber, rather than chemically absorbed.^2^ After exhaust dyeing, the dyed fiber is dried to remove the residual LA and fix the dyes.

The LA in the residual dyebath is recycled by evaporation, which can be reused for the successive dyeing. In comparison with conventional water dyeing, LA dyeing shortens the dyeing time, reduces energy consumption, avoids regulation of dyeing temperature, and saves auxiliary usage.^26,27^ In addition, cellulosic fibers are mercerized by the LA, enhancing the luster of the dyed fibers. Thus, in LA dyeing, simultaneous mercerization and coloration of fiber can be achieved. However, there are a few disadvantages of LA dyeing, such as low dye exhaustion and fixation rate, and poor colorfastness to washing and rubbing.^25,28,29^

In our previous work, cellulosic fiber was pre-treated with a 2,3-epoxypropyltrimethylammonium chloride cationic agent in an aqueous medium, and the cationic cellulosic fiber was subsequently dyed in LA dyebath,^30^ which considerably improved the dye exhaustion and fixation. Although the dye exhaustion increased by only 20%, the dye fixation rate reach almost 99%. To make the dyeing process fully waterless, the feasibility of cationic modification was also studied in an LA medium, and the cationization performance was similar to the fibers treated in an aqueous medium.^31^ In addition, LA and a combination of LA and water were used to efficiently wash off the unfixed dye, to further decrease water consumption in the dyeing process.^32^ In our next work, pretreatment to washing, the whole process was carried out in LA,^33^ which revealed that the dichlorotriazinyl group in the reactive dye was unstable and changed to a monochlorotriazinyl group during LA dyeing, which was possibly due to moisture present in LA, while the vinyl sulfone and monochlorotriazinyl reactive group were stable. Moreover, after drying, part of the vinyl sulfone or monochlorotriazinyl reactive group of the dye reacted with the hydroxyl groups of cellulose, resulting in covalent bonding. Other reactive dyes were hydrolyzed or did not change, *i.e.*, they remained in their reactive form.^33^ Thus, after drying, the unfixed dye washed off from the dyed fiber cannot be reused for dyeing.

Although the introduction of cationic groups in cellulosic fiber slightly promoted dye fixation, it also increases the potential of uneven color production because of the excessive increase in dye affinity. The dye fixation using a cationic fixing agent (CFA)/decamethylcyclopentasiloxane (D5) micro-emulsion system^2^ significantly increased the dye fixation (%) and the colorfastness to washing of the dyed fibers.

In industry, to produce a particular shade, normally two, three, or more dyes, are used to match the target color shade, and to achieve it the dyes used in the combination should have good compatibility in terms of dye exhaustion and fixation. In our previous work, we used only a single dye to investigate the dyeing performance in the LA medium.^32,33^ To make LA dyeing industrially viable, it is necessary to study the feasibility of dyeing with ternary dye mixtures. In the present work, three primary colors (C.I. Reactive Red 195, C.I. Reactive Yellow 145, and C.I. Reactive Blue 194) were used for the dyeing of ramie fibers in LA medium, since these three dyes showed good compatibility in aqueous dyeing. Dye compatibility with binary and ternary dyes mixtures was assessed by dye adsorption behavior. For improving the dye fixation (%), the aqueous and the LA dyed ramie fibers were after-treated with a CFA in a D5 medium.

## 2. Experimental

### 2.1 Materials

Pure ramie loose fibers were purchased from the Hunan Huasheng Zhuzhou Cedar Co., Ltd, China. C. I. Reactive Red 195 (R195), C. I. Reactive Yellow 145 (Y145), and C. I. Reactive Blue 194 (B194) were purchased from Shanghai Jiaying Chemical Company (China) and used as received. The chemical structures of these reactive dyes are presented in Table 1. Decamethylcyclopentasiloxane (D5) was purchased from Jiangxi Bluestar Xinghuo Silicones Company, China. Cationic fixing auxiliary (ALBAFIX® ECO), was supported by Huntsman Chemicals, USA. Liquid ammonia was purchased from Wuhan Niuruide Gas Company (China). Non-ionic detergent (Luton 500) was purchased from Dalton UK Company Ltd (China). All other reagents were analytical grade.

**Table 1:** Reactive dyes structures and molecular weights

Dye	Molecular structure	Molecular weight (g mol^−1^)
R195	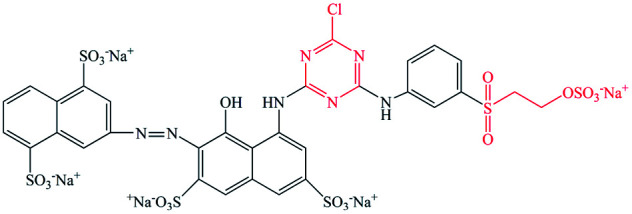	1136.3
Y145	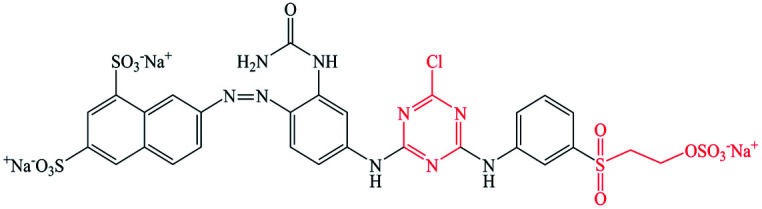	1026.3
B194	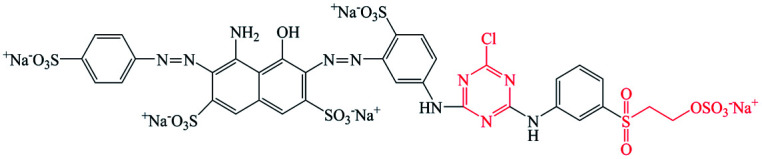	1308.1

### 2.2 Dyeing of ramie fiber

The dyeing of ramie fibers with 3% on the mass of fiber (o.m.f) of single dye (R195, Y145, and B194), 3% o.m.f of the binary dyes with a 1 : 1 ratio (R195, Y145, and B194), and 3% o.m.f of the ternary dyes with 1 : 1 : 1 ratio (R195, Y145, and B194) were at a liquor ratio of 50 : 1. For aqueous dyeing, the ramie fiber was immersed in dye solution at room temperature and then heated to 60 °C at a 2 °C min^−1^ heating rate, subsequently maintained at 60 °C for 10 to 60 min variation in an infrared radiation heated laboratory-rotary dyeing machine (Automatic Prototype, Model: A-12, AQUA, China). For LA dyeing, the LA dyebath was cooled to −40 °C using a refrigerant machine (FP40, JULABO Labortechnik GmbH, Germany). The ramie fiber was immersed in the LA dyebath for 1 to 10 min. After dyeing, the excess LA in the dyed ramie fiber was removed by placing in a hydro-extracting unit and centrifuged at 1400 revolutions per min (rpm) for 10 s. After which, the dyed ramie fiber was dried at 100 °C in an oven for 30 min to remove the residual LA in the dyed fiber and also to achieve dye-fixation, followed by soaping in a solution of 2 g L^−1^ non-ionic detergent at 95 °C for 15 min at a liquor ratio of 50 : 1.

### 2.3 Dye fixation treatment

The dye-fixation treatment was carried out in a laboratory-dyeing machine. The CFA solution was mixed with the D5 solvent and then the LA-dyed ramie fiber was soaked in it at a liquor ratio of 20 : 1 by varying various treatment parameters (Table 2). The fixing time refers to the duration of treatment at the target fixing temperature. After determination of the best dye fixation rate in a single factor variation, the factors and their levels for an L^9^ orthogonal experimental scheme are listed in Table 3, to optimize the fixing treatment conditions.

**Table 2:** Varied factors in dye fixation treatment

Variation	CFA mass (%, o.m.f)	Water mass (%, o.m.f)	Fixing time (min)	Fixing temperature (°C)
CFA	1–5	100	40	90
Water	4	100–500	40	90
Fixing time	4	100	10–50	90
Fixing temperature	4	100	40	60–100

**Table 3:** L^9^ orthogonal experimental scheme

Symbol	Process parameters	Unit	Level 1	Level 2	Level 3
A	CFA mass	%, o.m.f	3	4	5
B	Water mass	%, o.m.f	100	150	200
C	Fixing time	min	30	40	50
D	Fixing temperature	°C	80	90	100

### 2.4 Dye exhaustion percentage and fixation rate

The dye exhaustion percentage (*E*%) is the percentage amount of dye adsorbed by a fiber after dyeing with an applied dosage of dye. The dye mass in the residual dyebath was calculated from its standard curve (Tables S1 and S2†). The dye fixation rate (*F*%), the efficiency of the exhausted dye chemically bound on the fiber to the exhausted dye, was measured by measuring the color strength of the dyed sample before and after soaping. The *E*% and *F*% values were calculated from eqn (1) and (2).1
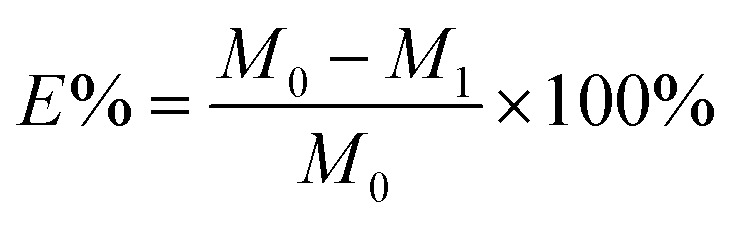
2
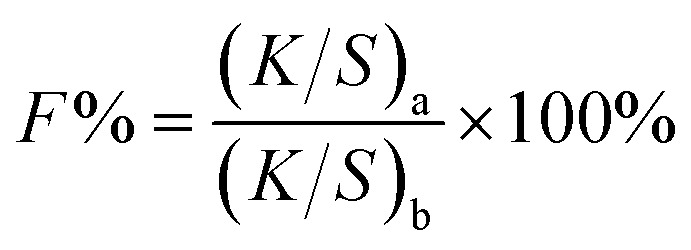
where *M*_0_ (g) and *M*_1_ (g) are the dye mass in the dyebath before and after dyeing, respectively, and (*K*/*S*)_b_ and (*K*/*S*)_a_ are the color strength of the dyed sample before and after soaping, respectively.

### 2.5 Characterization

The *K*/*S* values of each sample were measured using a reflectance spectrophotometer (CHN-Spec CS-650A, Hangzhou Color Spectrum Technology Company, China) and randomly recorded at 20 positions within the visible spectrum (*λ* = 400–700 nm) at an interval of 10 nm. The *K*/*S* value at the wavelength of the maximum absorption for a particular dye was measured, and the standard deviation of *K*/*S* (*σ*_*K*/*S*_) was calculated to assess the color uniformity. Lower values of the standard deviation are associated with a higher color uniformity of the dyed samples.^34^ The L*, a*, and b* values of the CIELab color space of each dyed sample were recorded with the spectrophotometer at 20 random positions, and the averages are reported here. The colorfastness to washing was measured as per ISO 105–C06:2010. Colorfastness to washing and the staining grade was measured by measuring the adjacent cotton fiber staining using a multi-fiber strip and comparing it with the ISO grayscale.

## 3. Results and discussion

### 3.1 Liquid ammonia dyeing with single reactive dye

**Fig. 1:**
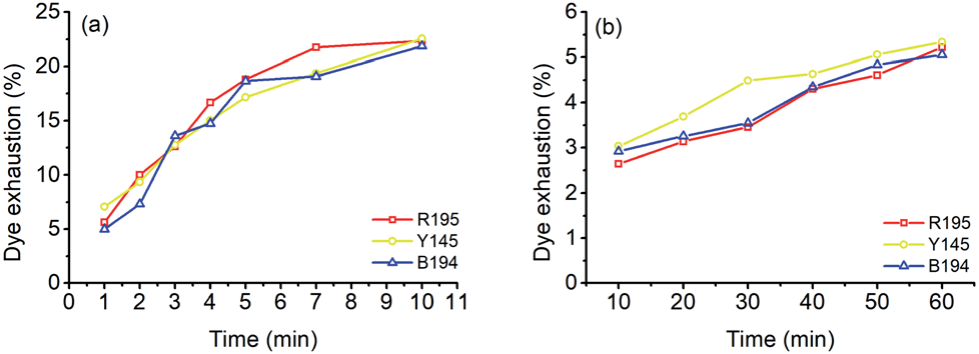
Dye exhaustion of ramie fiber dyeing using R195, Y145, and B194 in (a) LA and (b) water media.

In the LA dyeings of ramie fiber with a single reactive dye, the *E*% values are shown in Fig. 1a. The *E*% values of R195, Y145, and B194 proportionally increased with an increase in dyeing time. After 10 min of dyeing, the *E*% values were 22.3%, 22.5%, and 21.9% for R195, Y145, and B194, respectively, which were quite similar. The pores inside the ramie fiber are categorized as either small, medium, or large,^35^ which means that small molecules can easily enter all pores, but large molecules hardly enter small or medium pores. With LA treatment, the crystallinity of ramie fiber was reduced,^36^*i.e.* more amorphous regions were produced. However, it did not mean that the pore size of the ramie fiber was larger. LA contracted the pore size but increased the cumulatively accessible pore volume.^37^ During LA dyeing, the dyes adsorbed onto the fiber surface and then migrated into the interior of the fibers through the pores, and the pore size and dye molecular size were key parameters for dye diffusion.^38,39^ The molecular weights of R195, Y145, and B194 are 1136.3, 1026.3, and 1308.1 g mol^−1^ respectively (Table 1), which are quite close but still, the molecular weight of dyes is comparatively larger than other anionic dyes. Thus, the *E*% of these three dyes in LA dyeing were similar.

The *E*% values of ramie fibers in aqueous dyeing are presented in Fig. 1b. Similarly, the *E*% values of R195, Y145, and B194 exhibited uptake tendency with prolonged dyeing time, and the *E*% values increased around 3% to 5% in the dyeing time from 10 to 60 min. The *E*% in water dyeing slowly increased over the 60 min dyeing period, but it reached nearly dyeing equilibrium after 10 min in LA. In addition, it is worth noting that after 10 min dyeing, the *E*% values in the LA dyeing were about 8-fold higher compared to the aqueous dyeing. Since there was no salt added in LA dyeing (as opposed to the water dyeing process), the repulsive force between the cellulosic ramie fiber and the anionic reactive dyes was higher and exhibited a large inhibition rule for dye exhaustion.^40–42^ This implies that the dye adsorption rate in LA was very fast, and comparatively much higher than the aqueous dyeing.

**Fig. 2:**
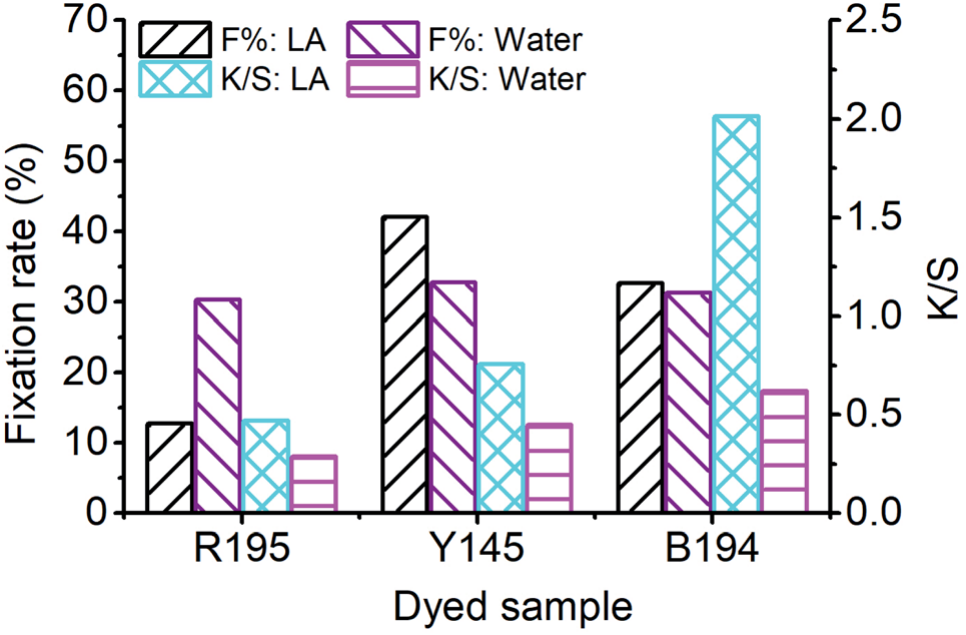
Dye fixation rate of ramie fiber dyeing with a single dye in LA and water media, and the *K*/*S* values of dyed samples after soaping.

After 10 and 60 min of LA dyeing and aqueous dyeing with a single dye, the dyed fibers were dried and soap-washed. The *F*% and *K*/*S* values (after soaping) of the dyed samples are displayed in Fig. 2, and the L*, a*, and b* values of the dyed samples are listed in Table 4. The *F*% values for LA dyeing were 12.77%, 42.04%, and 32.65% for R195, Y145, and B194, respectively, *i.e.*, the R195 had the worst fixation efficiency. In the aqueous dyeing without the addition of salt and soda ash, the *F*% values of the three dyes were in a narrow range of 30.32% to 32.77%. These results indicate that these three dyes in aqueous dyeing showed excellent compatibility as they exhibited similar dye fixation efficiency. It is worth noting that the *K*/*S* values are related to the dye concentration in the fiber when the fiber is completely dyed. Although the *F*% of R195 in LA dyeing was lower than that in aqueous dyeing, the *K*/*S* of the former was higher than that of the latter, due to the higher dye exhaustion.

**Table 4:** The L*, a*, and b* values of dyed samples (after soaping) and the color difference (Δ*E*) between LA and water dyeings

Dyed sample	LA			Water			Δ*E*
	L*	a*	b*	L*	a*	b*	
Single dye							
R195	74.88	20.19	−2.02	80.07	17.72	−2.79	5.80
Y145	82.95	5.92	30.67	85.81	1.11	24.23	8.53
B194	56.29	−6.35	−15.60	72.22	−7.33	−10.66	16.71

The color of dyed samples is described by L*, a*, and b* values, which are listed in Table 4. The a* (redness) value of R195, b* (yellowness) value of Y145, and b* (blueness) value of B194 of LA dyed fabrics were higher than the fabric dyed by the aqueous dyeing, which shows that the total dye mass absorbed in fiber was stronger in LA dyeing. In addition, the color difference (Δ*E*) between undyed and dyed fabric samples in the LA and the aqueous medium was calculated by eqn (3). The Δ*E* between the LA and the aqueous dyed fabrics with B194 was apparently larger than the Δ*E* values between the LA and aqueous dyed fabrics with the two other dyes (Table 4), suggesting that the fabric dyed with B194 had a higher color strength compared to the fabrics dyed with the other two dyes. The Δ*E* values were consistent with the *K*/*S* values determined by the eqn (3) presented in Fig. 2.3
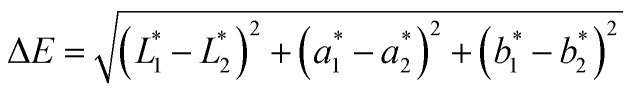
where 
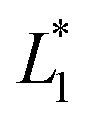
, 
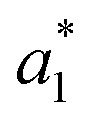
, and 
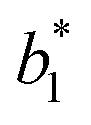
 are from the LA-dyed sample and 
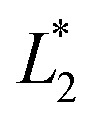
, 
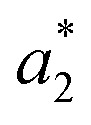
, and 
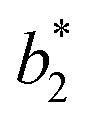
 are from the water-dyed sample. As shown in Fig. 2, for the R195, the aqueous dyed fabric showed better dye fixation (%) than the fabric dyed in the LA medium but for other two dyes, the LA dyed fabric showed the better dye fixation (%) compared to the aqueous dyed fabric.

### 3.2 Liquid ammonia dyeing with a binary dye mixture

**Fig. 3:**
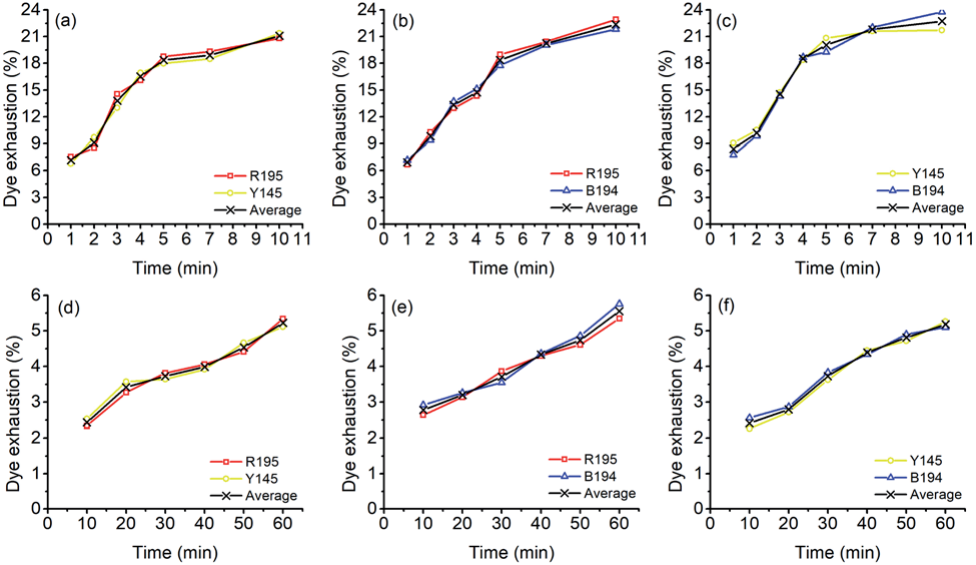
Dye exhaustion percentages in mixture dyeing of ramie fiber in LA with (a) a mixture of R195 and Y145, (b) R195 and B194, and (c) Y145 and B194, and in water with (d) a mixture of R195 and Y145, (e) R195 and B194, and (f) Y145 and B194.

The *E*% values of ramie fiber with a mixture of two dyes in LA are shown in Fig. 3a–c. In these mixed dyeing, the dye adsorption behaviors during LA dyeing were similar because the *E*% increased with increased dyeing time. This was described with a dramatic promotion of *E*% in 5 min and stabilization in the last 5 min. At the end of mixed dyeings (10 min), the *E*% values were 20.8% and 21.4%, 22.9% and 21.8%, and 21.7% and 23.8% for R195/Y145 (Fig. 3a), R195/B194 (Fig. 3b), and Y145/B194 (Fig. 3c), respectively. The results suggest that the compatibilities between two dye sets in LA were excellent, due to each dye showing similar substantivities, *i.e.* the attractions between the dye and the ramie fiber in the dyebath were close. Likewise, Fig. 3d–f displayed similar compatibilities between the two dyes in the aqueous dyeing.

During LA and aqueous dyeing with a mixture of two dyes, the dyes competed with each other, but both dyes in the mixture exhibited similar adsorption behavior. Furthermore, the total dye adsorption capacities (the average values in Fig. 3a–c) of ramie fiber in LA dyeing after 10 min were alike, which were 21.1%, 22.4%, and 22.7% for a mixture of R195/Y145, R195/B194, and Y145/B194, respectively. In addition, the total dye adsorption capacities (the average values in Fig. 3) among these dyeings in an aqueous medium after 60 min were close as well, which were 5.23%, 5.56%, and 5.18% for binary mixtures of R195 and Y145, R195 and B194, and Y145 and B194, respectively.

**Fig. 4:**
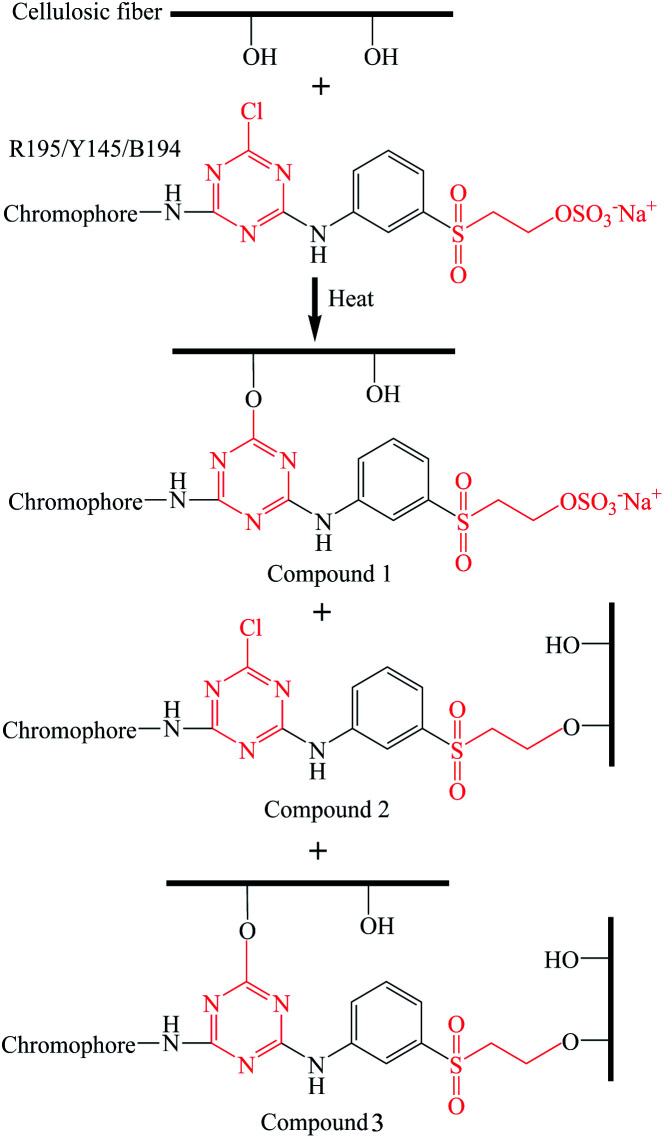
Dye fixation mechanism of ramie fiber with R195, Y145, and B194 dye.

The excess liquid ammonia was presented in the dyed sample once the dyed sample was taken out of the liquid ammonia dyebath. Thus the temperature of dyed ramie fiber was low, which froze the closed air and the moisture in the closed air was transferred to water and adsorbed in the dyed fiber. Thus, the adsorbed water mixed with liquid ammonia, and then ammonia water was produced, which changed the micro-circumstances of dye and fiber to be alkaline. During the drying at 100 °C, the residual liquid ammonia was evaporated, and the vinyl sulfone sulfate was transferred to vinyl sulfone group.^33^ Subsequently, some of the reactive dyes were chemically fixed with the ramie fiber by covalent bonding,^43^ as shown in Fig. 4. The covalent bonds were formed between the hydroxyl groups of ramie fiber and monochlorotriazinyl groups, vinyl sulfone groups, or both.

**Fig. 5:**
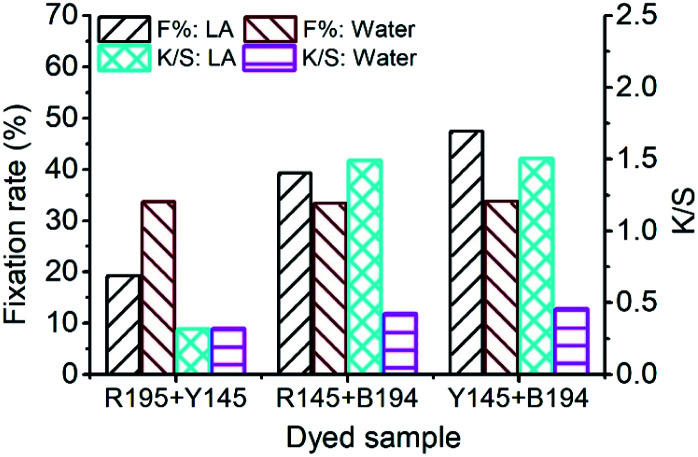
Dye fixation rates of ramie fiber dyeing using binary dyes in LA and water media and the *K*/*S* values of dyed samples after soaping.

The *F*% and *K*/*S* values (after soap-washing) of the dyed sample using binary dyes are shown in Fig. 5. The *E*% of each dye in each binary LA dyeing was close, but the fixing property of each dye was different, resulting in a difference in the *F*% of each binary LA dyeing. Since the *F*% of R195 in LA dyeing was the poorest among the three dyes (Fig. 2), it lowered the *F*% of binary R195/Y145 dyeing and reduced the *F*% of binary R195/B94 dyeing. The highest *F*% was for the binary Y145/B194 dyeing, which was 47.40%. By comparison, the binary dyeing in an aqueous medium after 60 min showed *F*% values of 33.68%, 33.39%, and 33.77% for the binary dyeings of R195/Y145, R195/B194, and Y145/B194, respectively, because of the similar fixation efficiencies exhibited by these three dyes.

In the *K*/*S* values of dyed samples, the color strength of the dyed fiber by binary R195/Y145 dyeing in LA decreased to the level of the aqueous dyeing, owing to its low *F*%. Conversely, the color strength of the dyed sample by binary dyeing of R195/Y145 was about three times stronger compared to dyeing in water. Similarly, it was also about 3 times stronger for the binary dyeings of Y145/B194 in LA compared to that in water.

### 3.3 Liquid ammonia dyeing with ternary dye mixture

**Fig. 6:**
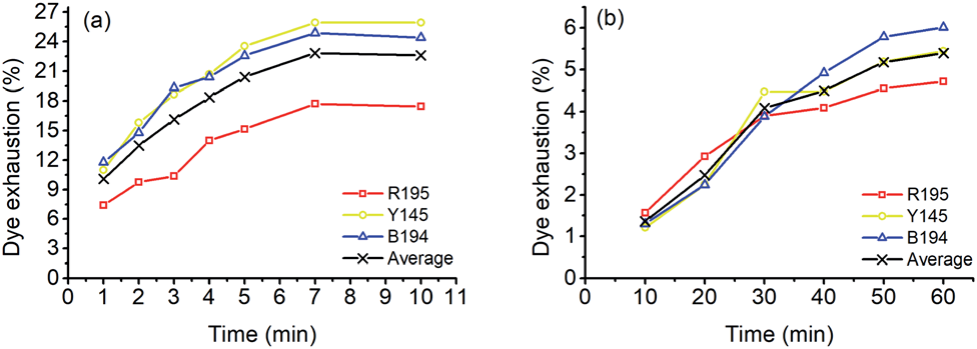
Dye exhaustion percentages in mixture dyeing of ramie fiber using a combination of R195, Y145, and B194 and the average in (a) LA and (b) water.

The *E*% values of ternary dyeing of ramie fiber in LA and water media were displayed in Fig. 6. It shows that the compatibility of the three dyes in aqueous dyeing was better compared to the LA dyeing. In LA dyeing (Fig. 6a), the *E*% of the three dyes increased with an increase in the dyeing time. However, the *E*% of R195 was lower than that of Y145 and B194, which was 17.45% for 10 min dyeing, compared to the Y145 and B194, which were 25.92% and 24.42%, respectively. In the aqueous dyeing, the *E*% of dyes increased gradually as well, and the three dyes showed a similar exhaustion tendency during dyeing, despite a slight difference in *E*% in dyeing for 60 min. Of the three dyes, R195 exhibited the poorest competition for dye absorption on ramie fiber, which was 4.72% compared to 5.45% for Y145 and 6.02% for B194. The total dye adsorption capacities (the average values in Fig. 6) of ramie fiber dying in LA for 10 min and water for 60 min were 22.60% and 5.40%, respectively. In summary, the total dye adsorption capacities in single (Fig. 1), binary (Fig. 3), and ternary (Fig. 6) dyeing in both LA and water were stable.

**Fig. 7:**
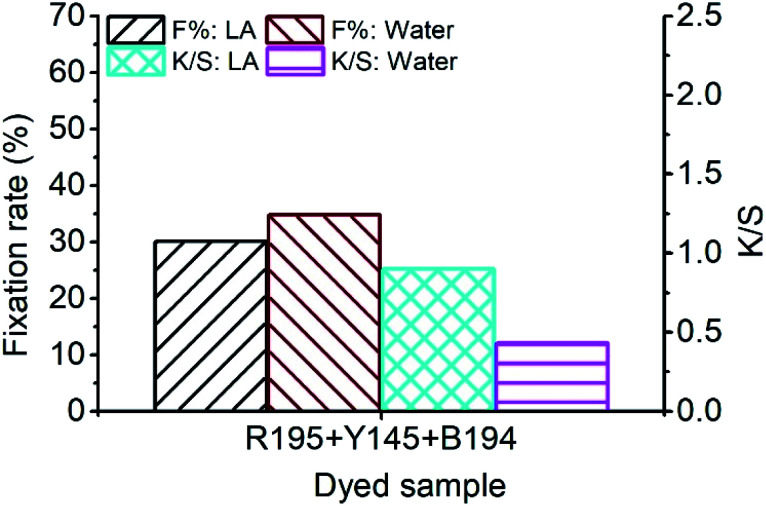
Dye fixation rate of ramie fiber dyeing using ternary dyes in LA and water media and the *K*/*S* values of dyed samples after soaping.

In Fig. 7, the *F*% of ramie fiber dyed with ternary dyes in LA and water was 30.10% and 34.74%, respectively. The poor fixing property of R195 in LA dyeing lowered the *F*% of the ternary dyeing in LA (Fig. 2), and the *F*% of the ternary dyeing in water was still stable, which was near the level of single dyeing (Fig. 2) and binary dyeing (Fig. 5). Although the *F*% of water dyeing using ternary dyes was higher than that in LA dyeing, the *E*% in LA dyeing was 4 times higher than that in water dyeing, which resulted in a relatively higher total dye fixation in LA dyeing, accompanied by a relative high *K*/*S* value, *i.e.* stronger color strength of the dyed sample.

Therefore, based on the low *F*% of single, binary, and ternary dyeing in LA, it can be concluded that the dye fixation by drying is unsuitable for LA dyed ramie fiber with reactive dyes, although it is an easy and simple method. In other words, it is essential to apply a high efficiency of dye fixation to improve the dyeing performance of LA dyeing.^2^

### 3.4 Cationic fixation treatment of dyed ramie fiber

**Fig. 8:**
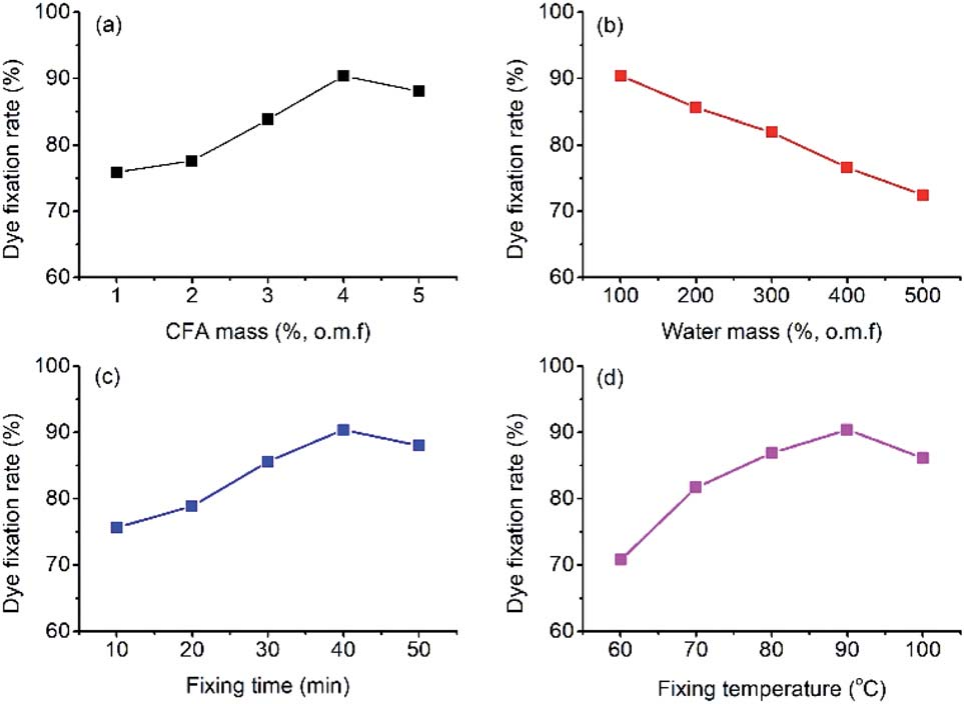
Dye fixation rate of CFA/D5 micro-emulsion treatment with variations of (a) CFA mass, (b) water mass, (c) fixing time, and (d) fixing temperature.

The ramie fiber was dyed with a ternary mixture of reactive dyes (1% o.m.f of R195, 1% o.m.f of Y145, and 1% o.m.f of B194) at a liquor ratio of 1 : 50 at −40 °C for 10 min. After dyeing and LA extraction, the dyed ramie fiber was treated in the CFA/D5 micro-emulsion, and the dye fixation (%) calculated by eqn (2) are displayed in Fig. 8. The highest dye fixation was achieved at 4% o.m.f CFA, 100% o.m.f water, 40 min fixing time, and 90 °C fixing temperature. It is worth noting that in the water mass variation (Fig. 8b), further decrease of water mass to 50% o.m.f produced uneven color.

**Fig. 9:**
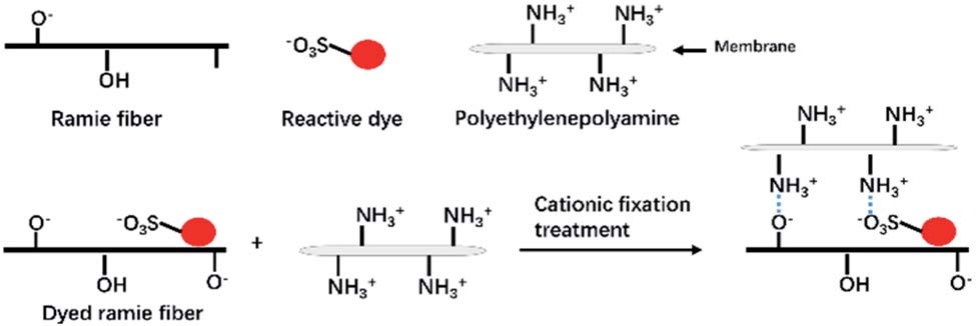
Mechanism of polyethylene polyamine fixed unfixed reactive dye in ramie fiber.

The quaternary ammonium cationic groups (–NH_3_^+^) of polyethylene polyamine (the main component of the CFA) cross-linked with the sulfonic groups of reactive dyes (dye–SO_3_^−^) and cellulosate anions of fiber (cellulose–O^−^). Besides, a layer of CFA was formed on the surface of the fiber, which interfered with the unfixed dye removal in the washing process. Therefore, the cationic fixation treatment not only improved the dye fixation (%), but also improved the colorfastness to washing.^44^ The dye fixation mechanism is shown in Fig. 9.

**Fig. 10:**
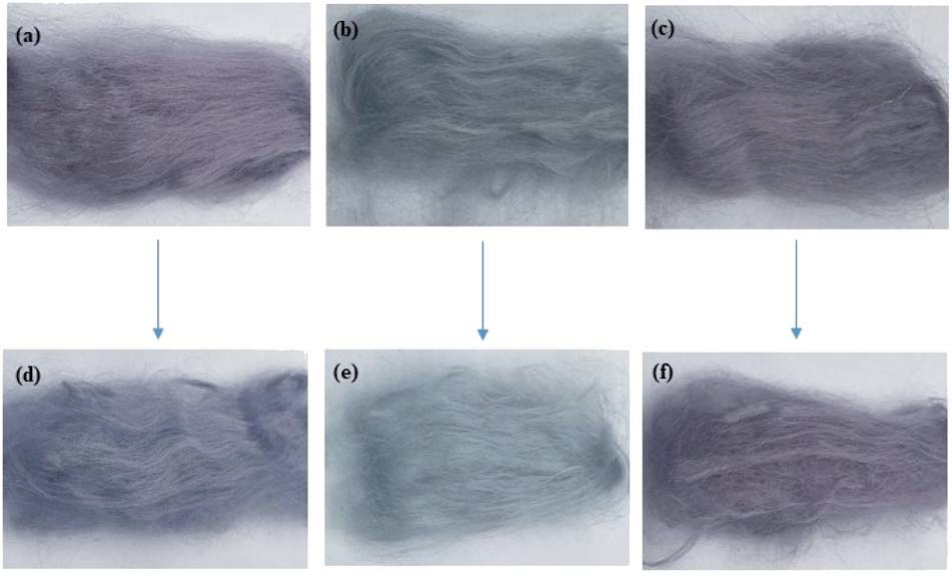
The dyed ramie fiber (a) without soaping and without fixation treatment, (b) without fixation treatment but with soaping, (c) with CFA/D5 micro-emulsion fixation treatment and soaping, and their samples after wash fastness test are (d), (e), and (f), respectively.

To explore the optimum conditions of dye fixation with CFA/D5 micro-emulsion, an L^9^ orthogonal experimental scheme was designed, and the treatment conditions with the relevant *F*% values are listed in Table 5. The highest *F*% achieved was 96.78% for conditions of 5% o.m.f CFA mass, 100% o.m.f water mass, 40 min fixing time, and 80 °C fixing temperature, which is the optimum conditions for dye fixation. In Fig. 10, the CFA fixation treatment showed a crucial contribution to dye fixation, and its color shade (Fig. 10c) was similar to what was before the treatment (Fig. 10a); whereas, the color of dyed ramie fiber after soaping (Fig. 10b) became lighter. The color properties of these samples, including L*, a*, and b* values, *K*/*S*, and color uniformity (*σ*_*K*/*S*_) are listed in Table 6. The CFA treatment on dye fixation of LA-dyed ramie fiber exhibited a great effect.^2,45^ With the dye-fixation treatment, the Δ*E* value of samples a and c (in Fig. 9) was 3.47, but without treatment it increased to 17.70, *i.e.*, without the CFA treatment, many adsorbed dyes in the dyed ramie fibers were desorbed in the soaping process. Meanwhile, the poor color shade of sample b hints that dye fixation by drying for LA dyeing is unsuitable. Besides, the *K*/*S* value of the dyed ramie fiber with CFA treatment is 2.31, which is similar to that before CFA treatment (2.39).

**Table 5:** The L^9^ orthogonal array of treatment conditions and their *F*% values

Number	CFA mass (%, o.m.f)	Water mass (%, o.m.f)	Fixing time (min)	Fixing temperature (°C)	*F*% (%)
1	3	50	30	80	90.19
2	4	50	40	90	94.17
3	5	50	50	100	89.38
4	3	100	50	90	91.10
5	4	100	30	100	88.80
6	5	100	40	80	96.65
7	3	150	40	100	83.13
8	4	150	50	80	91.37
9	5	150	30	90	78.84

**Table 6:** Color properties of dyed ramie fibers with and without fixation treatment

Sample^a^	L*	a*	b*	Δ*E* (sample − sample)	*K*/*S*	*σ*_*K*/*S*_
Sample a	49.31	3.58	−7.97	—	2.39	0.23
Sample b	65.26	−3.43	−5.52	17.60 (b − a)	0.80	0.05
Sample c	52.59	2.79	−7.17	3.47 (c − a)	2.31	0.07
Sample d	52.81	0.88	−7.42	4.45 (d − a)	1.81	0.07
Sample e	65.97	−3.57	−5.79	0.77 (e − b)	0.74	0.05
Sample f	54.18	0.35	−7.59	2.94 (f − c)	1.71	0.08

a Samples are displayed in Fig. 10.

After washing as per the colorfastness to washing standard, the dyed sample without soaping and without fixation treatment (Fig. 10a) showed considerable change in color after the washing test (Fig. 10d) with Δ*E* of 4.45, and the *K*/*S* value reduced to 1.81 from 2.39. The washing fastness (staining) of the washed sample was Grade 3–4. The sample without fixation treatment but with soaping showed an ignorable color change (Fig. 10e) after washing with a Δ*E* of 0.77, and the *K*/*S* value only reduced to 0.74 from 0.80, because the unfixed dyes were almost removed after soaping, resulting in its washing fastness (staining) Grade 4–5. For the sample that had a fixation treatment and soaping, the color shade after washing slightly changed (Fig. 10f) with a Δ*E* of 2.94 and *K*/*S* value of 1.71 reduced from 2.31, and its washing fastness (staining) was Grade 4–5. The color change of this sample after wash fastness testing was ignorable by visual detection; thus, the wash fastness (fade) of this sample was Grade 4–5. Thus, finally, all the dyed samples showed color uniformity, and the soaping and washing processes improved their color uniformity owing to the decreased *σ*_*K*/*S*_ values.^31^

### 3.5 Color triangle of dyed ramie fibers

In preparing the color triangle of dyed ramie fibers, the total dye mass was 5% o.m.f. The dyed ramie fibers in LA and water using R195, Y145, and B194 with various dye mass ratios before fixation treatment (without soaping) are shown in Fig. S3 and S4,† respectively, and their corresponding L*, a*, and b* values of each sample with dye mass ratio are listed in Tables 7 and 8, respectively. The color shades of dyed samples exhibited a range of colors. The color of dyed ramie fiber by LA dyeing showed a darker shade, compared to the sample that was aqueous dyed at the same dye mass ratio. These samples were treated by the dye fixation process with optimal conditions and soaped. The treated dyed fibers are displayed in Fig. S5,† and their color properties are depicted in Table 9. The results showed that the color of all dyed samples slightly changed owing to the high dye fixation rate. The standard deviation of *K*/*S* values (*σ*_*K*/*S*_) was low, which means that all dyed samples have good color uniformity. Therefore, the results indicated the optimum conditions of dye fixation were appropriate for LA-dyed ramie fiber.

**Table 7:** The L*, a*, and b* values and *K*/*S* values of the dyed ramie fiber using R195, Y145, and B194 in LA with various dye mass ratios

Sample number	Dye mass ratio (R195 : Y145 : B194)	L*	a*	b*	*K*/*S*
1	1 : 0 : 0	54.36	45.54	−7.91	3.57
2	0.8 : 0.2 : 0	54.03	44.38	2.94	3.64
3	0.8 : 0.1 : 0.1	45.80	25.80	−11.41	4.13
4	0.8 : 0 : 0.2	42.21	20.84	−19.20	4.84
5	0.6 : 0.4 : 0	56.61	40.38	11.18	2.71
6	0.6 : 0.3 : 0.1	46.92	20.61	−3.70	3.45
7	0.6 : 0.2 : 0.2	46.38	13.54	−10.60	3.26
8	0.6 : 0.1 : 0.3	46.52	11.52	−14.50	3.16
9	0.6 : 0 : 0.4	40.81	12.03	−20.30	4.72
10	0.4 : 0.6 : 0	59.66	33.92	20.19	2.17
11	0.4 : 0.5 : 0.1	44.82	12.10	0.80	3.38
12	0.4 : 0.4 : 0.2	45.12	8.99	−2.97	3.03
13	0.4 : 0.3 : 0.3	41.68	6.78	−8.26	4.10
14	0.4 : 0.2 : 0.4	37.99	5.84	−12.59	5.31
15	0.4 : 0.1 : 0.5	37.17	5.45	−15.65	5.70
16	0.4 : 0 : 0.6	37.99	5.33	−19.44	5.56
17	0.2 : 0.8 : 0	63.35	29.97	31.43	2.75
18	0.2 : 0.7 : 0.1	52.20	8.68	12.81	2.77
19	0.2 : 0.6 : 0.2	48.43	3.17	5.47	2.30
20	0.2 : 0.5 : 0.3	42.90	0.99	0.36	3.40
21	0.2 : 0.4 : 0.4	41.93	−0.45	−5.89	3.84
22	0.2 : 0.3 : 0.5	40.67	−1.97	−8.11	4.40
23	0.2 : 0.2 : 0.6	40.33	−1.86	−11.86	4.77
24	0.2 : 0.1 : 0.7	37.30	−0.55	−15.08	6.03
25	0.2 : 0 : 0.8	35.75	−0.27	−18.37	7.21
26	0 : 1 : 0	75.12	15.78	53.67	3.11
27	0 : 0.9 : 0.1	56.36	−4.05	24.01	3.39
28	0 : 0.8 : 0.2	51.11	−7.42	13.44	3.15
29	0 : 0.7 : 0.3	47.73	−8.55	7.08	2.85
30	0 : 0.6 : 0.4	45.71	−9.03	2.17	3.48
31	0 : 0.5 : 0.5	41.03	−8.94	−2.43	5.07
32	0 : 0.4 : 0.6	39.14	−8.32	−5.20	5.89
33	0 : 0.3 : 0.7	39.43	−7.94	−8.68	6.03
34	0 : 0.2 : 0.8	36.61	−6.60	−11.72	7.46
35	0 : 0.1 : 0.9	36.33	−5.91	−13.94	7.77
36	0 : 0 : 1	34.40	−4.35	−17.05	9.13

**Table 8:** The L*, a*, and b* values and *K*/*S* of the dyed ramie fibers using R195, Y145, and B194 in water with various dye mass ratios

Sample number	Dye mass ratio (R195 : Y145 : B194)	L*	a*	b*	*K*/*S*
1	1 : 0 : 0	73.69	25.71	−5.57	0.61
2	0.8 : 0.2 : 0	74.60	18.70	9.94	0.50
3	0.8 : 0.1 : 0.1	68.58	7.20	−4.49	0.63
4	0.8 : 0 : 0.2	66.83	5.00	−13.41	0.73
5	0.6 : 0.4 : 0	78.45	13.74	19.09	0.59
6	0.6 : 0.3 : 0.1	72.14	2.61	6.28	0.53
7	0.6 : 0.2 : 0.2	68.44	0.10	−0.74	0.57
8	0.6 : 0.1 : 0.3	63.72	−0.22	−8.62	0.88
9	0.6 : 0 : 0.4	62.46	−0.04	−15.48	1.06
10	0.4 : 0.6 : 0	80.52	10.93	23.45	0.62
11	0.4 : 0.5 : 0.1	72.87	1.08	11.19	0.64
12	0.4 : 0.4 : 0.2	69.90	−1.46	5.43	0.48
13	0.4 : 0.3 : 0.3	69.51	−3.48	0.73	0.55
14	0.4 : 0.2 : 0.4	66.50	−3.50	−4.15	0.74
15	0.4 : 0.1 : 0.5	66.29	−4.09	−8.39	0.82
16	0.4 : 0 : 0.6	64.92	−3.61	−13.97	0.98
17	0.2 : 0.8 : 0	79.76	11.41	33.79	1.04
18	0.2 : 0.7 : 0.1	71.74	−1.31	18.49	0.97
19	0.2 : 0.6 : 0.2	68.11	−4.35	12.14	0.95
20	0.2 : 0.5 : 0.3	68.18	−5.87	6.64	0.74
21	0.2 : 0.4 : 0.4	64.53	−6.79	2.42	0.88
22	0.2 : 0.3 : 0.5	63.50	−7.23	−1.68	1.02
23	0.2 : 0.2 : 0.6	63.40	−7.13	−5.75	1.08
24	0.2 : 0.1 : 0.7	61.65	−6.66	−10.04	1.28
25	0.2 : 0 : 0.8	61.60	−5.67	−15.44	1.36
26	0 : 1 : 0	81.92	5.30	37.16	1.04
27	0 : 0.9 : 0.1	71.91	−3.39	24.71	1.24
28	0 : 0.8 : 0.2	67.71	−6.98	16.55	1.19
29	0 : 0.7 : 0.3	66.58	−8.09	12.26	1.07
30	0 : 0.6 : 0.4	63.89	−9.11	8.22	1.08
31	0 : 0.5 : 0.5	62.27	−9.59	4.21	1.14
32	0 : 0.4 : 0.6	62.20	−10.03	0.59	1.20
33	0 : 0.3 : 0.7	61.10	−10.06	−3.08	1.37
34	0 : 0.2 : 0.8	59.91	−9.76	−6.53	1.54
35	0 : 0.1 : 0.9	59.56	−9.21	−10.74	1.64
36	0 : 0 : 1	61.03	−8.10	−15.56	1.55

**Table 9:** The L*, a*, and b* values, *K*/*S* values, *σ*_*K*/*S*_ values, and *F*% values of the LA-dyed ramie fibers treated by the dye fixation process

Sample number	Dye mass ratio (R195 : Y145 : B194)	L*	a*	b*	*K*/*S*	*σ*_*K*/*S*_	*F*% (%)
1	1 : 0 : 0	53.91	38.73	−11.32	3.15	0.12	88.24
2	0.8 : 0.2 : 0	53.95	37.61	−1.87	3.02	0.13	82.97
3	0.8 : 0.1 : 0.1	46.49	22.21	−13.10	3.81	0.24	92.25
4	0.8 : 0 : 0.2	43.92	17.93	−20.03	4.05	0.20	83.68
5	0.6 : 0.4 : 0	53.78	30.94	4.91	2.61	0.39	96.31
6	0.6 : 0.3 : 0.1	48.42	17.59	−6.18	3.02	0.13	87.54
7	0.6 : 0.2 : 0.2	48.40	10.72	−11.27	2.79	0.17	85.58
8	0.6 : 0.1 : 0.3	48.10	8.97	−14.93	2.84	0.13	89.87
9	0.6 : 0 : 0.4	42.87	9.27	−20.44	4.18	0.16	88.56
10	0.4 : 0.6 : 0	57.18	29.52	13.17	1.96	0.12	90.32
11	0.4 : 0.5 : 0.1	47.06	9.67	−1.36	2.87	0.27	84.91
12	0.4 : 0.4 : 0.2	45.65	6.58	−4.00	2.91	0.43	96.04
13	0.4 : 0.3 : 0.3	41.59	4.64	−9.63	3.96	0.26	96.59
14	0.4 : 0.2 : 0.4	40.06	3.66	−13.60	4.78	0.21	90.02
15	0.4 : 0.1 : 0.5	39.55	3.17	−16.61	5.09	0.31	89.30
16	0.4 : 0 : 0.6	39.05	2.86	−19.93	5.46	0.35	98.20
17	0.2 : 0.8 : 0	61.80	24.20	26.35	2.46	0.12	89.45
18	0.2 : 0.7 : 0.1	53.88	6.50	10.61	2.24	0.22	80.87
19	0.2 : 0.6 : 0.2	47.95	1.41	3.59	2.14	0.21	93.04
20	0.2 : 0.5 : 0.3	43.24	−0.63	−1.15	3.27	0.16	96.18
21	0.2 : 0.4 : 0.4	44.61	−2.09	−6.63	3.35	0.27	87.24
22	0.2 : 0.3 : 0.5	42.67	−3.64	−8.91	4.06	0.34	92.27
23	0.2 : 0.2 : 0.6	40.65	−3.48	−12.94	4.33	0.39	90.78
24	0.2 : 0.1 : 0.7	38.30	−2.86	−15.89	5.93	0.61	98.34
25	0.2 : 0 : 0.8	37.52	−2.89	−18.73	6.89	0.51	95.56
26	0 : 1 : 0	69.64	10.75	43.42	2.93	0.63	94.21
27	0 : 0.9 : 0.1	57.26	−4.20	21.58	2.83	0.08	83.48
28	0 : 0.8 : 0.2	52.05	−7.68	12.15	2.75	0.21	87.30
29	0 : 0.7 : 0.3	49.65	−8.94	6.42	2.52	0.31	88.42
30	0 : 0.6 : 0.4	47.86	−9.80	1.79	3.07	0.18	88.22
31	0 : 0.5 : 0.5	42.88	−9.89	−3.17	4.57	0.18	90.14
32	0 : 0.4 : 0.6	41.21	−9.78	−6.11	5.38	0.55	91.34
33	0 : 0.3 : 0.7	40.67	−9.12	−9.34	5.72	0.33	94.86
34	0 : 0.2 : 0.8	38.74	−8.53	−12.60	6.89	0.66	92.36
35	0 : 0.1 : 0.9	37.68	−8.02	−14.77	7.62	0.62	98.07
36	0 : 0 : 1	35.87	−6.95	−17.75	9.00	0.61	98.58

## 4. Conclusions

The dyeing of ramie fiber with a mixture of ternary dyes in the LA medium was investigated and compared to aqueous dyeing. The dye exhaustion (%) of ramie fiber dyed with a single dye was almost 22% and 5% for R195, Y145, and B194 in LA and water media, respectively. The dye adsorption behaviors indicated that R195, Y145, and B194 dyes have high compatibility for LA and water dyeing. The fixation rates of these three dyes are similar to each other in the range of 30–32% in water dyeing, but distinguishing differences within a range of 12–42% in LA dyeing, stating the fixation by drying for LA dyeing was unsatisfactory. For LA dyeing with binary and ternary dye mixtures, the three dyes exhibited satisfactory adsorption behaviors and compatibility. The CFA/D5 micro-emulsion was applied to improve dye fixation after LA dyeing with the ternary dye mixture, and the dye fixation rate improved to 96.65% with optimal dye fixation conditions (5% o.m.f of CFA mass, 100% o.m.f of water mass, 40 min of fixing time, and 80 °C of fixing temperature). The dyed ramie fiber with fixing treatment displayed a high color uniformity and excellent wash fastness with Grade 4–5 of color staining and Grade 4–5 of color fade. The colorfully dyed ramie fibers in the color triangle identified that many color shades of ramie fiber can be prepared. This work demonstrates that the LA dyeing method could be a viable and industrially feasible sustainable dyeing method for the dyeing of ramie fiber.

## Conflicts of interest

There are no conflicts to declare.

## Supplementary Material

RA-012-D2RA03288K-s001
