# Supplementary material for: Sustainable dyeing of ramie fiber with ternary reactive dye mixtures in liquid ammonia
Source: RSC Adv. 2022 Jul 1;12(30):19253–64. doi: 10.1039/d2ra03288k (PMC9247807; doi:10.1039/d2ra03288k)
Supplement: RA-012-D2RA03288K-s001 [file RA-012-D2RA03288K-s001.pdf]

Electronic Supplementary Material (ESI) for RSC Advances.
This journal is © The Royal Society of Chemistry 2022

Supplementary Information

Sustainable dyeing of ramie fiber with ternary reactive dye mixtures in liquid ammonia

Bo Gao[‡1,2], Xiaolong Huang[‡1,3], Tiancheng Jiang[1,3], Md. Nahid Pervez[4], Wenju Zhu[1,3], Mohammad Mahbubul Hassan[5], Yingjie Cai[1,3*], Vincenzo Naddeo[4*]

[1]Hubei Provincial Engineering Laboratory for Clean Production and High-Value Utilization of Bio-based Textile Materials, Wuhan Textile University, Wuhan, 430200 China

[2]College of Art and Design, Wuhan Textile University, Wuhan 430200, China

[3]Engineering Research Centre for Clean Production of Textile Dyeing and Printing, Ministry of Education, Wuhan Textile University, Wuhan, 430200 China

[4]Sanitary Environmental Engineering Division (SEED), Department of Civil Engineering, University of Salerno, Fisciano, 84084 Italy

[5]Fashion, Textiles and Technology Institute (FTTI), University of the Arts London, 20 John Prince's Street, London W1G 0BJ, United Kingdom

[‡]These authors contributed equally to the work

* Corresponding authors:

Email: yingjiecai@wtu.edu.cn (Yingjie Cai), vnaddeo@unisa.it (Vincenzo Naddeo)

The light absorbance curves of R195, Y145, B194 from 380-800 nm are displayed in Fig. S1, and the maximum absorption wavelengths of R195, Y145, and B194 are 542 nm, 418 nm, and 600 nm, respectively. The standard curves of these three dyes, i.e. a plot of dye concentration VS light absorbance, are shown in Fig. S2, and the correlative linear equations are listed in Table S1. The Eq. S2, Eq. S4, and Eq. S9 were used to work out the dye concentration in the R195, Y145, and B194 dye solutions respectively. In calculation of each dye concentration in binary mixture and in ternary mixture, the correlative equations are listed in Table S2. The color triangles of dyed ramie fibers using R195, Y145, and B194 in LA and in water with various dye mass ratios are shown in Fig. S3 and Fig. S4, respectively. The color triangle of the LA-dyed ramie fibers treated by the dye fixation process is shown in Fig. S5.

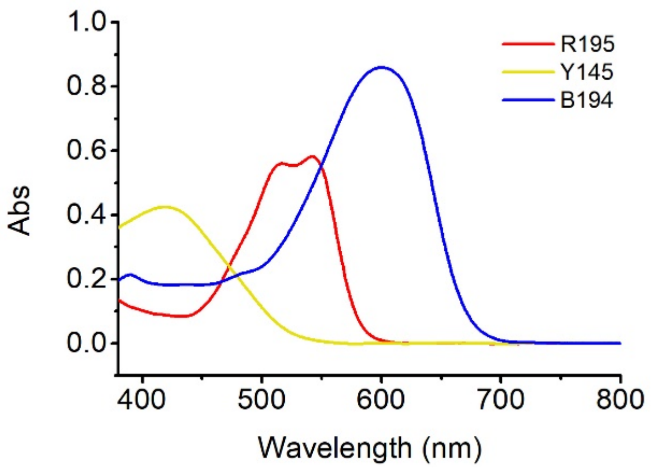

Fig. S1 Light absorbance curves of R195, Y145, and B194

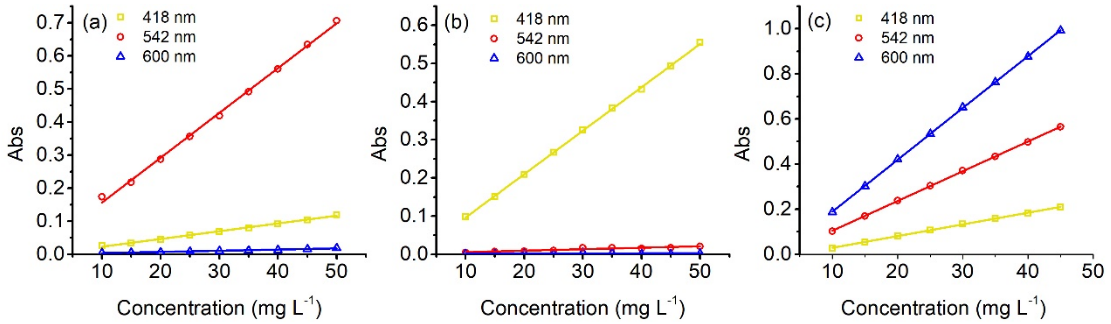

Fig. S2 The standard curves of (a) R195, (b) Y145, and (c) B194 at 418 nm, 542 nm, and 600 nm

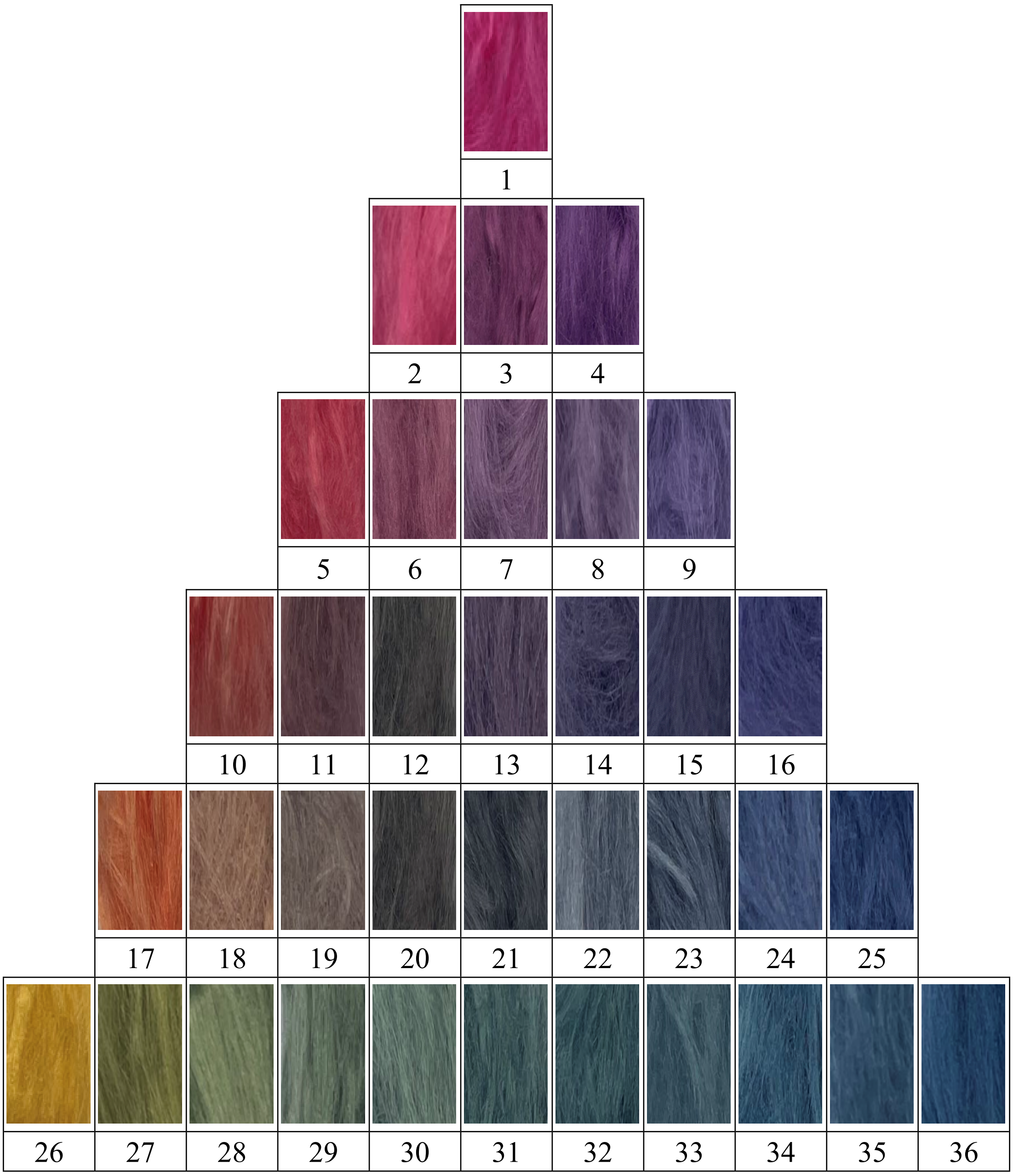

Fig. S3 Color triangle of dyed ramie fibers using R195, Y145, and B194 in LA with various dye mass ratios

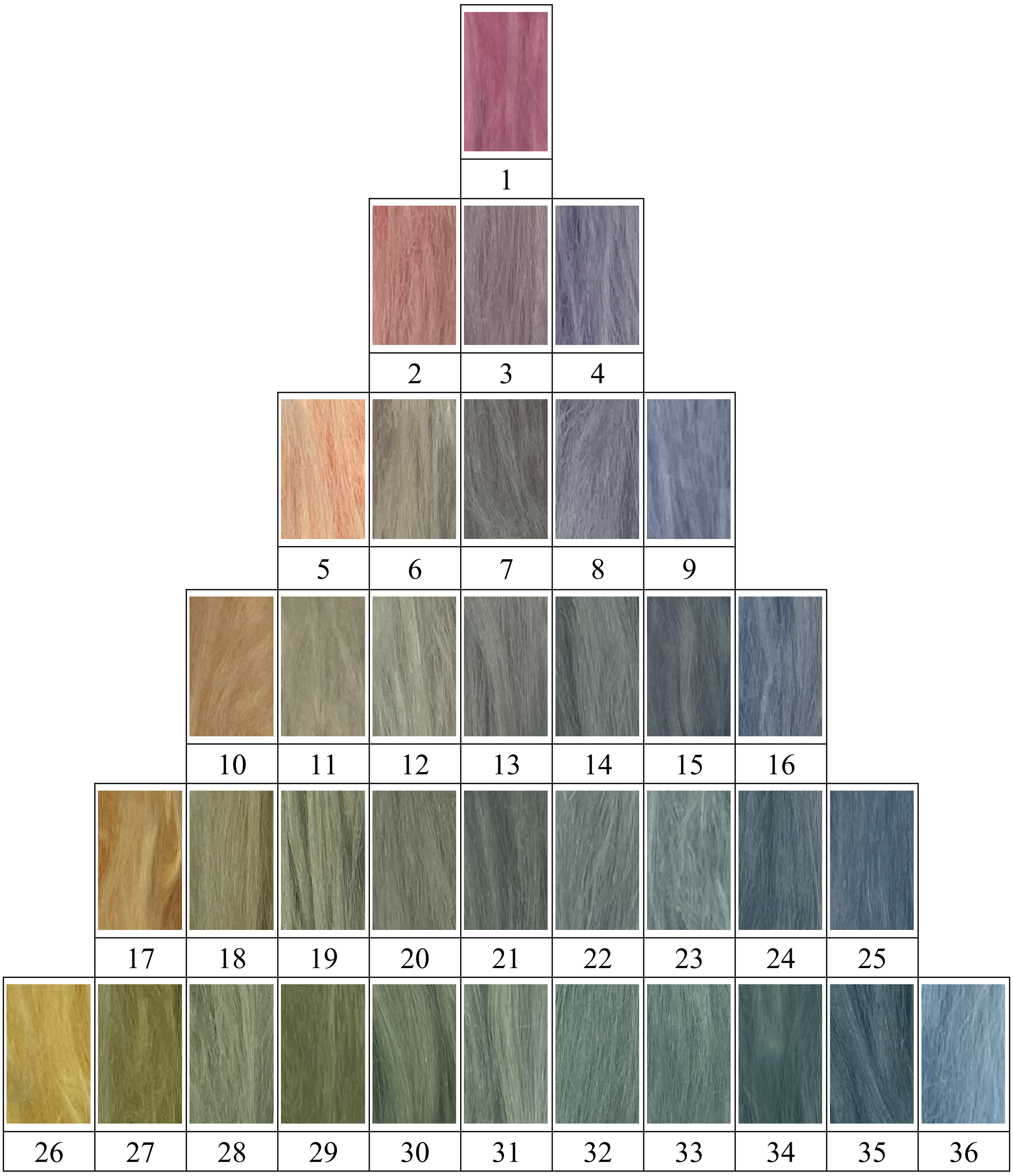

Fig. S4 Color triangle of the dyed ramie fibers using R195, Y145, and B194 in water with various dye mass ratios

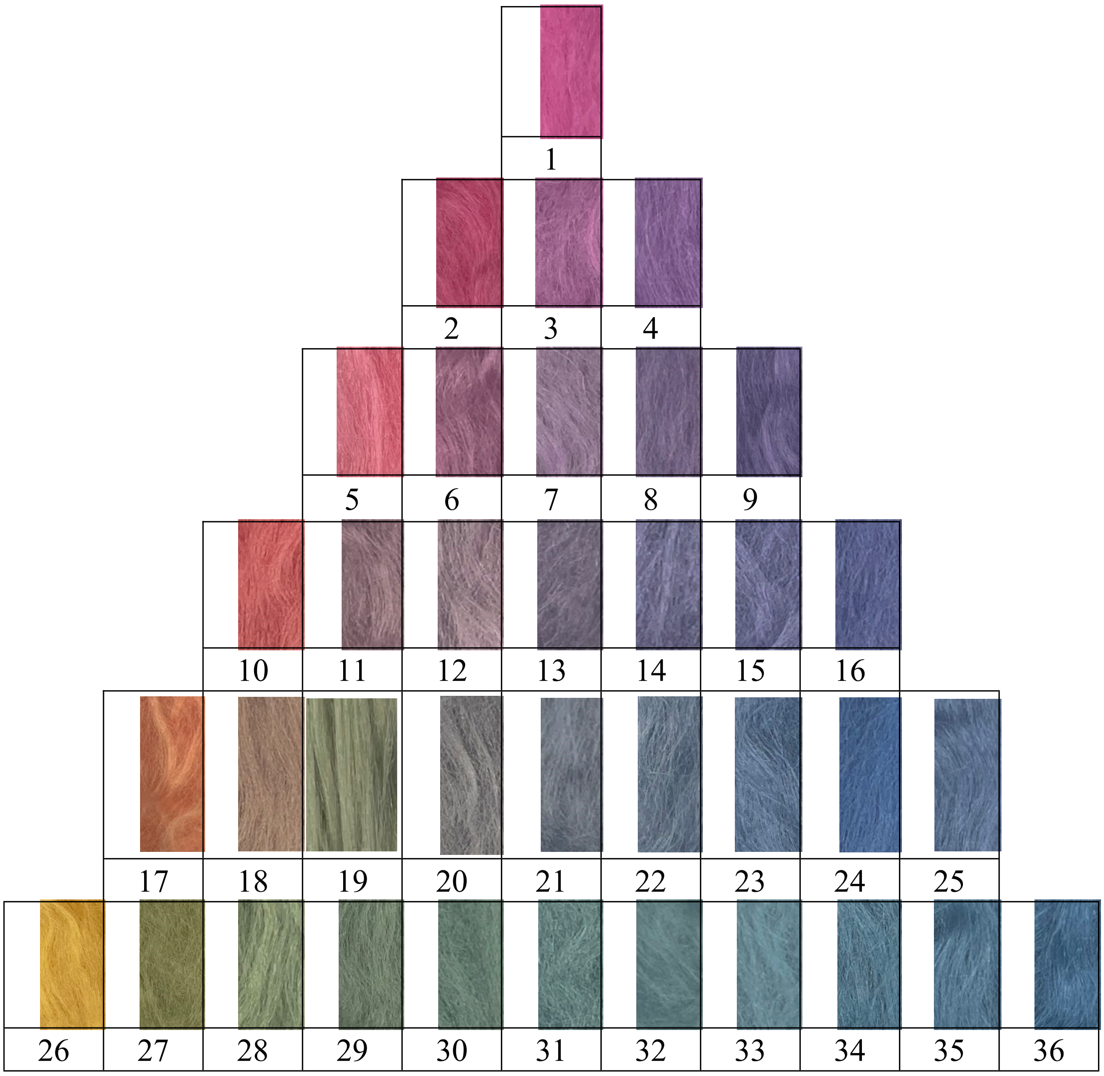

Fig. S5 Color triangle of the LA-dyed ramie fibers treated by the dye fixation process

Table S1 The linear equation of the standard curves of R195, Y145, and B194

Dye	Wavelength (nm)	Equation	Equation number	R^2
R195	418	$A_{R\text{-}418} = 0.00235\mathbf{C_R} - 0.00101$	S1	0.9958
	542	$A_{R\text{-}542} = 0.01355\mathbf{C_R} + 0.02065$	S2	0.9979
	600	$A_{R\text{-}600} = 0.0003523\mathbf{C_R} - 0.0002922$	S3	0.9788
Y145	418	$A_{Y\text{-}418} = 0.01137\mathbf{C_Y} - 0.01758$	S4	0.9997
	542	$A_{Y\text{-}542} = 0.00038667\mathbf{C_Y} + 0.00129$	S5	0.8703
	600	$A_{Y\text{-}600} = 0.000018667\mathbf{C_Y} - 0.00128$	S6	0.02106
B194	418	$A_{B\text{-}418} = 0.00518\mathbf{C_B} - 0.02336$	S7	0.9998
	542	$A_{B\text{-}542} = 0.01319\mathbf{C_B} - 0.02756$	S8	0.9999
	600	$A_{B\text{-}600} = 0.02298\mathbf{C_B} - 0.0411$	S9	1.0000

Table S2 Equations of binary mixture and ternary mixture

Dye	Equation	Equation number
Binary mixture		
R195+Y145	$A_{RY-542} = A_{R-542} + A_{Y-542}$	S10
	$= 0.01355\mathbf{C}_R + 0.0003867\mathbf{C}_Y + 0.02194$	
	$A_{RY-418}=A_{R-418}+A_{Y-418}$	S11
	$= 0.00235\mathbf{C}_R + 0.01137\mathbf{C}_Y - 0.01859$	
R195+B194	$A_{RB-542} = A_{R-542} + A_{B-542}$	S12
	$= 0.01355\mathbf{C}_R + 0.01319\mathbf{C}_B - 0.00691$	
	$A_{RB-600} = A_{R-600} + A_{B-600}$	S13
	$= 0.0003523C_R + 0.02298\mathbf{C}_B - 0.0413922$	
Y145+B194	$A_{YB-418} = A_{Y-418} + A_{B-418}$	S14
	$=0.01137\mathbf{C}_Y + 0{,}00518\mathbf{C}_B - 0.04094$	
	$A_{YB-600} = A_{Y-600} + A_{B-600}$	S15
	$=0.00001867\mathbf{C}_Y +0.02298\mathbf{C}_{\mathbf{B}} - 0.04238$	
Ternary mixture		
R195+Y145	$A_{RYB-542} = A_{R-542} + A_{Y-542} + A_{B-542}$	S16
+B194	$= 0.01355\mathbf{C}_R + 0.0003867\mathbf{C}_Y + 0.01319\mathbf{C}_B -0.00562$	
	$A_{RYB-418} = A_{R-418}+A_{Y-418}+A_{B-418}$	S17
	$= 0.00235\mathbf{C}_R + 0.01137\mathbf{C}_Y + 0.00518\mathbf{C}_B -0.04195$	
	$A_{RYB-600} = A_{R-600}+A_{Y-600}+A_{B-600}$	S18
	$= 0.0003523\mathbf{C}_R + 0.00001867\mathbf{C}_Y + 0.02298\mathbf{C}_B - 0.0426722$	
